# Use of Savitzky–Golay Filter for Performances Improvement of SHM Systems Based on Neural Networks and Distributed PZT Sensors

**DOI:** 10.3390/s18010152

**Published:** 2018-01-08

**Authors:** Mario A. de Oliveira, Nelcileno V. S. Araujo, Rodolfo N. da Silva, Tony I. da Silva, Jayantha Epaarachchi

**Affiliations:** 1 Department of Electrical and Electronic, Mato Grosso Federal Institute of Technology, Cuiabá 78005-200, Brazil; rodolfonogueirasilva@gmail.com (R.N.d.S.); tony.silva@cba.ifmt.edu.br (T.I.d.S.); 2 Institute of Computing, Federal University of Mato Grosso, Cuiabá 78060-900, Brazil; nelcileno@ic.ufmt.br; 3 Centre of Excellence in Engineered Fiber Composites, Faculty of Engineering and Surveying, University of Southern Queensland (USQ), Toowoomba, QLD 4667, Australia; jayantha.epaarachchi@usq.edu.au

**Keywords:** SHM, electromechanical impedance, fuzzy ARTMAP network, probabilistic neural network, artificial intelligence, Euclidean distance, piezoelectricity, pattern recognition

## Abstract

A considerable amount of research has focused on monitoring structural damage using Structural Health Monitoring (SHM) technologies, which has had recent advances. However, it is important to note the challenges and unresolved problems that disqualify currently developed monitoring systems. One of the frontline SHM technologies, the Electromechanical Impedance (EMI) technique, has shown its potential to overcome remaining problems and challenges. Unfortunately, the recently developed neural network algorithms have not shown significant improvements in the accuracy of rate and the required processing time. In order to fill this gap in advanced neural networks used with EMI techniques, this paper proposes an enhanced and reliable strategy for improving the structural damage detection via: (1) Savitzky–Golay (SG) filter, using both first and second derivatives; (2) Probabilistic Neural Network (PNN); and, (3) Simplified Fuzzy ARTMAP Network (SFAN). Those three methods were employed to analyze the EMI data experimentally obtained from an aluminum plate containing three attached PZT (Lead Zirconate Titanate) patches. In this present study, the damage scenarios were simulated by attaching a small metallic nut at three different positions in the aluminum plate. We found that the proposed method achieves a hit rate of more than 83%, which is significantly higher than current state-of-the-art approaches. Furthermore, this approach results in an improvement of 93% when considering the best case scenario.

## 1. Introduction

Structural integrity monitoring using Non-Destructive Evaluation (NDE) methods have become more popular in recent years as they can be applied to a wider range of applications. Expensive and critical infrastructures must be monitored to achieve their designed lifetime and avoid premature failures [1]. To monitoring the conditions of infrastructure, NDE methods have been created, which are based on different techniques, such as: acoustic emission, Eddy current, radiography, thermography, shearography, Lamb waves, and electromechanical impedance [2]. Many mechanical, civil, and aerospace engineering structures are subjected to severe environmental conditions and different types of loads. Over the years, these structures suffer from degradation and can be damaged without prior warning. A strict preventive maintenance process can prevent major damage and ensure the smooth operation of the infrastructure. However, this process significantly increases the operating costs of infrastructure. For this reason, Structural Health Monitoring (SHM) techniques have been extensively studied to increase safety and reduce maintenance costs.

Electromechanical Impedance (EMI) and Lamb waves techniques have been widely used in recent years for SHM research [3,4,5,6,7,8,9,10,11,12]. Both methodologies require the knowledge of the response signals of the undamaged structure. This response is called the baseline, which is used as a reference for the identification of possible structural damage through the comparison of responses under normal and unknown operational conditions. Historically, signal analysis was usually carried out through multivariate analysis, sensor data fusion, and machine learning approaches [13]. Recently, the nearest neighbor algorithm, which is a machine learning approach, has been proposed to analyze data obtained from PZT (Lead Zirconate Titanate) sensors to investigate different types of damage: such as breaks, corrosion, cracks, impact damage, delamination, disunity, and breaking of fibers [14]. The above-mentioned earlier method was based on guided waves. Related to the subject of damage identification in SHM systems using the EMI technique, other suitable methods have been introduced and explored in previous studies [10,12]. Recently, researchers proposed a method to identify crack-length-related resonances in acoustic emission waveforms for monitoring structural health [15]. They conducted several experiments based on the fatigue analysis in order to obtain the acoustic waveforms based on Acoustic Emission (AE). The AE waveforms were analyzed and classified into three types based on the similar nature in both the time and frequency domains. The results confirmed that the local crack resonance phenomenon was due to the interaction between the AE waveform and fatigue crack, with this phenomenon related to the crack length.

The EMI principle was first applied to SHM systems in 1994 [16]. Subsequently, several SHM systems on EMI have been developed and used in a variety of applications. Recently Baptista et al. [17] conducted an experimental study of effects of temperature on the electrical impedance of the PZT sensors used in the EMI technique. In another study, Castro et al. investigated the feasibility of using low-cost piezoelectric sensors to identify partial discharges in the mineral insulating oil of power transformers [18]. In a recent study, Kim and Park [19] proposed a novel method for estimation of the early age strength of concrete by introducing an artificial neural network algorithm to process the dynamic response measurements of concrete structures. They used both electromechanical impedances and guided ultrasonic waves signals, which were obtained from an embedded piezoelectric sensor module. In this sense, Hoshyarmanesh et al. [20] proposed a low-cost, compact, and portable transceiver for exploring periodic structural health monitoring of a rotary structure using the EMI technique. The authors highlighted that the compactness of the proposed system is an essential requirement for rotary structures when compared with bulky, heavy, and expensive impedance analyzers. The authors also claimed that their system is time-efficient, although they did not provide any reference value for comparison. More advances in the identification of structural damage based on the EMI can be found in previous studies [20,21,22,23,24,25].

Methods based on Neural Networks (NN) have been widely proposed in the context of SHM applications [19,26,27,28]. Recently, new classes of artificial networks, such as Probabilistic Neural Network (PNN) and Fuzzy ARTMAP Network (FAN), have been proposed based on their considerable performances, such as improved accuracy rates and reduced time consumption. More details of the methods based on PNN and FAN being applied to identify structural damage in SHM systems can be found in references [10,11,12,29,30,31] and references [32,33], respectively. Ali et al. [34] highlighted that the application of the Simplified Fuzzy ARTMAP Network (SFAN) in large-scale systems or online-based methods has significantly enhanced the accuracy of predictions and reduced processing times. They have shown that SFAN has the best performance in terms of training/testing speed when compared to the Back-Propagation network. De Oliveira and Inman [35] proposed a method based on SFAN, which they applied to structural damage assessment in composite structures using the EMI. In the same context, they [36] presented a comparative analysis of SFAN and PNN for identifying structural damage growth. The authors have used the EMI in the time-domain to address analysis in terms of the influence of certain SFAN setup parameters. As a result, they showed that SFAN parameters have a substantial influence on the SFAN performance. Hence, they proposed a method for choosing SFAN optimal parameters automatically [37].

Unfortunately, there are many grey areas in signal processing algorithms for PZT data analysis. Furthermore, there are no details regarding the integration of Savitzky–Golay (SG) filters with both first and second derivatives in SFAN/PNN via the EMI technique. This paper proposes a novel and reliable strategy for damage detection with higher confidence levels, which is based on neural networks. The performance of the proposed methodology will be experimentally investigated using an aluminum plate containing three attached PZT patches. The structural damage will be simulated as metallic nuts, which will be bonded at three different positions.

In summary, the two main objectives of this present study are:

- develop an improved method based on the EMI technique that is relatively more efficient in terms of damage detection rates when compared to state-of-the-art approaches; and,
- develop a method that uses a short dataset for training neural networks, which would be a great advantage for practical applications in SHM systems.

The remainder of the paper is organized as follows. Firstly, the main theoretical fundamentals are addressed. Secondly, the proposed method is presented, followed by the experimental results and discussion. Finally, the paper highlights the final remarks of the proposed approach.

## 2. Theoretical Fundamentals

### 2.1. Electromechanical Impedance

The basic principle behind the Electromechanical Impedance (EMI) technique is the application of high frequency structural excitations (typically greater than 20 kHz) through the surface-bonded PZT transducers, before measuring the impedance of structures by monitoring the current and voltage applied to the PZT. Changes in the PZT’s impedance indicate changes in the structure, which subsequently can be used to interpret damage status. For the last two decades, extensive SHM research has been performed to investigate the damage status of structural components that are used in mechanical, aerospace, and civil engineering applications. These techniques have been extended to be used in piezoelectric sensor diagnostics, concrete cure monitoring, and biomedical applications. In practice, a source with variable frequency results in the structure vibrating at its natural frequencies, with these responses used to estimate the Frequency Response Function (FRF) for computing EMI. The presence of damage will change these natural frequencies, causing shifts in frequency and amplitude. Statistical metrics compute the differences between the healthy structural condition (baseline) and the unknown conditions using EMI to detect either the presence or absence of structural damage.

**Figure 1:**
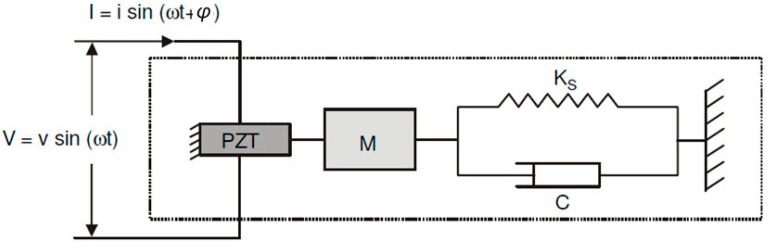
Electromechanical coupling between the PZT patch and the host structure.

The technique based on the EMI for SHM applications was initially proposed by Liang et al. [16] and is an important form of the Non-Destructive Evaluation (NDE) method. This technique uses PZT transducers that are glued onto the monitored structure. When subjected to a mechanical stimulus, the PZT transducer converts mechanical energy into electrical energy (sensor). When subjected to an electric stimulus, the PZT transducer converts electric energy into mechanical energy (actuator). Hence, when considering a linear PZT transducer, the EMI behavior can be described as follows [38]:

(1) $$\left[\begin{array}{c} S\\ D \end{array}\right]=\left[\begin{array}{c} s^{E}\;d_{t}\\ d\;\varepsilon^{t} \end{array}\right]\;\left[\begin{array}{c} T\\ E \end{array}\right]$$

where S represents the mechanical deformation, T is the mechanical stress, E is the electric field, D is the charge density, s is the complacency, d is the piezoelectric deformation constant, and ε is the permissiveness. The first line of the matrix describes the inverse effect of the PZT, while the second one describes the direct effect. Figure 1 shows the structure, including the PZT, which is represented by an electromechanical model of the mass-spring type with a single degree of freedom [16]:

In Figure 1, M represents the mass, $K_{s}$ is the elastic spring constant, and C is the damping coefficient. If the transducer is excited through a sine wave source V with magnitude v and angular frequency ω, then it will produce a current I of magnitude i and phase φ. Following this, the electrical impedance of the PZT ($Z_{E}$(ω) can be determined, as follows [16]:

(2) $$Z_{E}\left(\omega \right)=\frac{V}{I}=\frac{1}{j\omega a}{\left({\bar{\varepsilon }}_{33}{}^{T}-\frac{Z\left(\omega \right)}{Z\left(\omega \right)+Z_{A}\left(\omega \right)}d_{3x}^{2}{\hat{Y}}_{\mathrm{xx}}^{E}\right)}^{-1},$$

where $Z_{A}\left(\omega \right)$ and Z(ω) represent the mechanical impedances for the transducer and monitored structure, respectively. In Equation (2), ${\bar{\varepsilon }}_{33}{}^{T},$ ${\hat{Y}}_{\mathrm{xx}}^{E},$ $d_{3x}^{2},$ a and j represent the dielectric constant, Young’s modulus, transverse piezoelectric charge coefficient, geometric constant and imaginary unit, respectively. From Equation (2), we can see that any variation in terms of the structural impedance will cause changes in the electrical impedance of the PZT patch, which subsequently causes changes in the EMI signatures.

It is important to mention that the frequency range of the EMI, which exhibits greater sensitivity and repeatability in detecting structural damage, depends on various structural factors, including geometry, mass, boundary conditions, and other characteristics. The most advantageous concepts behind the EMI techniques are related to their use of high frequencies in order to reduce the interference from global conditions, such as environmental vibrations. In addition, excitations using high frequencies make the wavelength relatively small, allowing for the detection of small damage, such as cracks, delamination, and loosened screws. In many cases, such faults are missed by methods that use low frequency excitation signals. Recommended reading related to using the EMI technique in the SHM field can be found in previous studies [7,16,39].

### 2.2. Savitzky–Golay Filter

The Savitzky–Golay (SG) smoothing filter is considered as a type of Finite Impulse Response (FIR) digital filter, which is represented by polynomial equations. Based on the least squares method [40], the SG filter is typically used to smooth a noisy signal whose frequency range of the signal without noise is large. In this type of application, the SG filter performs better than the standard FIR filters because these tend to attenuate a significant portion of high frequencies of the signal along with noise. Although the SG filter is more effective in preserving relevant high frequency components, SG filters are less effective in removing high level noises in a signal. The particular formulation of the SG filter preserves moments of higher orders much better than other methods. As a consequence, the widths and amplitudes of the peaks for the desired signals tend to be preserved [41].

The SG filter can be understood by considering an arbitrary degree *d* of the polynomial fit and to an arbitrary N length of the noise data N-dimensional vector x. Assuming that N = 2M + 1 and is odd for a sequence of M, a natural number, points symmetrical to each side of *x*_0_, as follows [41]:

(3) $$x={\left[x_{-M},\ldots ,x_{-1},x_{0},x_{1},\ldots ,x_{M}\right]}^{T}.$$

The N data samples of x can be fitted by a polynomial, on *d*, of order *d* (0 ≤ *d* ≤ M), according to the following equation:

(4) $${\hat{x}}_{m}=c_{0}+c_{1}m+\cdots +c_{d}m^{d}\;-M\leq m\leq M.$$

where ${\hat{x}}_{m}$ represents the *m^th^* smoothed data sample. The coefficients $c_{i}$ are real numbers and must be determined optimally such that the corresponding polynomial curve best fits the given data [41]. Then, we define *d* + 1 polynomial basis vectors represented by $s_{i}$ (*i* = 0, 1, …, *d*) [41]:

(5) $$s_{i}\left(m\right)=m^{i}-M\leq m\leq M.$$

The corresponding S matrix (N × (*d* + 1)) is set to have $s_{i}$ as a column:

(6) $$S=\left[s_{0},s_{1},\ldots ,s_{d}\right].$$

Hence, the smoothed values can be organized in a vector as follows:

(7) $$\hat{x}=Bx=\sum_{i=0}^{d}c_{i}s_{i}.$$

However, the value *y*_0_ *=* ${\hat{x}}_{0}$ is given in terms of the center of the filter **b**_0_ according to below:

(8) $$y_{0}=b_{0\;}^{T}x=\sum_{m=-M}^{M}b_{0}\left(m\right)x_{m}.$$

The N-dimensional vector x can be shifted of *n* instants of time as follows:

(9) $$x\;\to \left[x_{n-M},\ldots ,x_{n-1},x_{n},x_{n+1},\ldots ,x_{n+M}\right]{\;}^{T}.$$

A SG filter with length N and order *d* for smoothing noise in a sequence x(n) at the steady state can be represented by [41]:

(10) $$y\left(n\right)=\sum_{m=-M}^{M}b_{0}\left(-m\right)\;x\left(n-m\right).$$

By differentiating Equation (10) *i* times, we obtain the generic form of the filter output [40]:

(11) $$y^{i}\left(n\right)=i!\sum_{m=-M}^{M}g_{i}\left(-m\right)x\left(n-m\right)i=0,1,\;\ldots ,d.$$

The use of derivatives is usually interesting when we want to removed offsets of the signals during the pre-processing phase [42]. Usually, the first derivative removes the systematic offsets of signal, while the second derivative can eliminate linear variations.

### 2.3. Neural Networks

**Figure 2:**
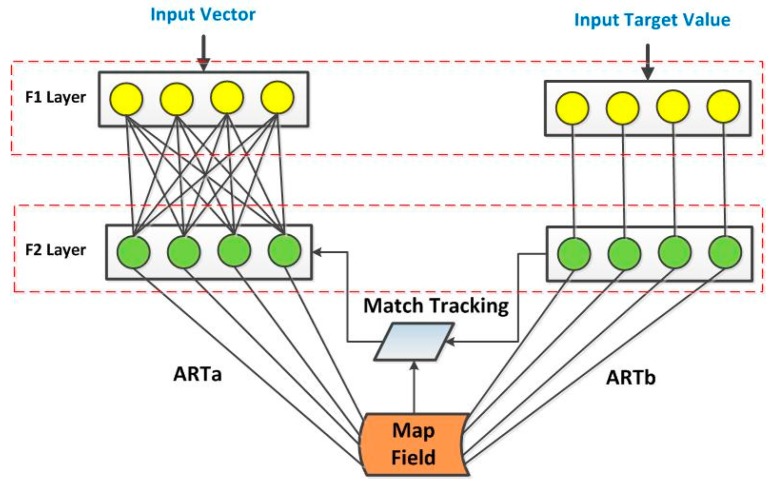
General diagram for the Fuzzy ARTMAP Network (FAN) network.

This approach explores two different neural networks: Simplified Fuzzy ARTMAP Network (SFAN) and the Probabilistic Neural Network (PNN). The FAN architecture has a supervised learning network [43]. The Fuzzy ARTMAP Network (FAN) algorithm has two modules (ARTa and ARTb) that are connected by the MAP Field module. Under this approach, the ARTa module is fed with EDs values computed from the smoothed signals (SG, first and second derivatives), while the ARTb module has four recognition categories associated with four structural conditions (H, D1, D2, and D3). Figure 2 summarizes the architecture of the FAN. The FAN parameters are briefly described as follows. Briefly, the choice parameter (α) defines the degree of interference in the selection of the most representative neurons of the weight vector. The training rate (β) controls the speed at which the neural network learns. The vigilance parameter (ρ) looks for differences among input patterns that are responsible for creating new categories through similarity tests. The match tracking (ε) checks if there is a match between the input (ARTa module) and output (ARTb module). Unlike the FAN algorithm, the SFAN algorithm takes a further step when compared to FAN in reducing the computational overhead and architectural redundancy, which slows down the training of the network [43]. Further details about SFAN/FAN algorithms can be explored in previous studies [43,44].

**Figure 3:**
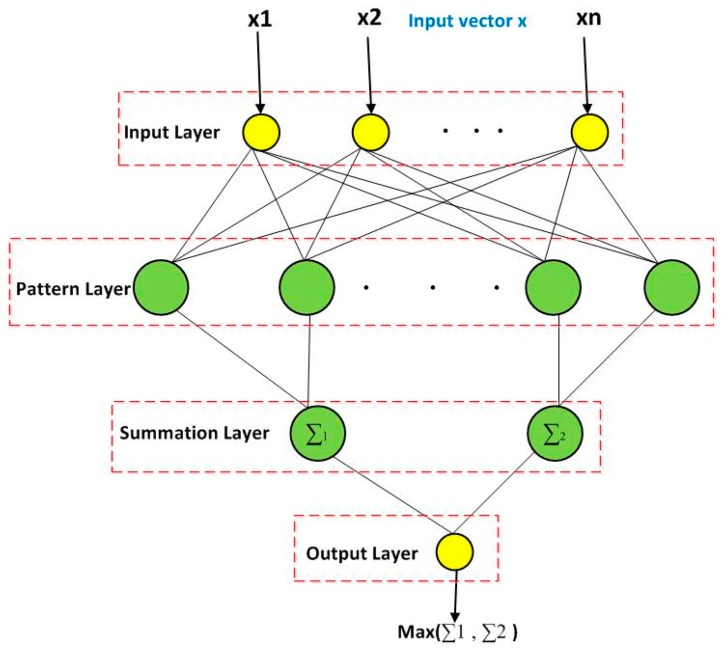
General diagram for the Probabilistic Neural network (PNN).

A Probabilistic Neural Network (PNN) [45] was introduced. The PNN consists of four layers of neurons: input layer, pattern layer (hidden layer), summation layer, and output layer. The structure of PNN is illustrated in Figure 3. Further details about PNN can be obtained from a previous study [45].

## 3. Proposed Method

**Figure 4:**
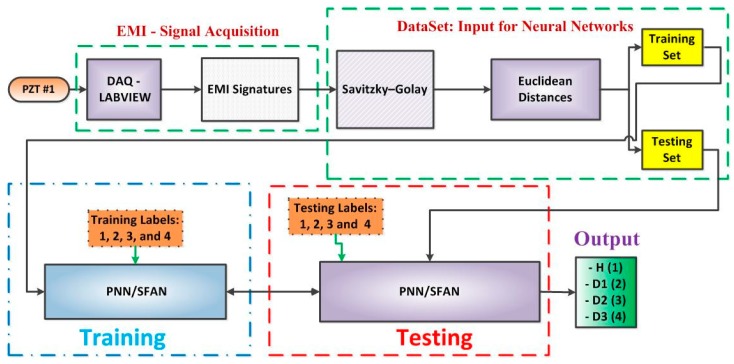
General diagram for the proposed methodology for PZT#1 and Savitzky–Golay filter.

The proposed methodology for detecting structural damage is presented here. The methodology consists of three phases: acquisition of the EMI signals, forming datasets, and conducting the damage detection module. In Phase 1, the impedance signals were obtained based on the EMI technique. For that, three PZTs (PZT#1, PZT#2, and PZT#3) were attached to the monitored structure. Damage scenarios were simulated by attaching a metallic nut at three different positions (D1, D2 and D3). In Phase 2, datasets are formed by smoothing all of the impedance signals through the Savitzky–Golay filter (SG), SG with first, and SG with second derivatives. From the smoothed signals, Euclidean Distances (EDs) are computed. In Phase 3, the detection module is carried out using PNN/SFAN algorithms. This module is responsible for classifying the presence (D1, D2 and D3) or absence of structural damage (Healthy (H)). For example, Figure 4 sums up the whole methodological procedure for PZT #1 after the application of the SG filter. This procedure is similar for other PZTs.

### 3.1. Signal Acquisition Based on EMI

**Figure 5:**
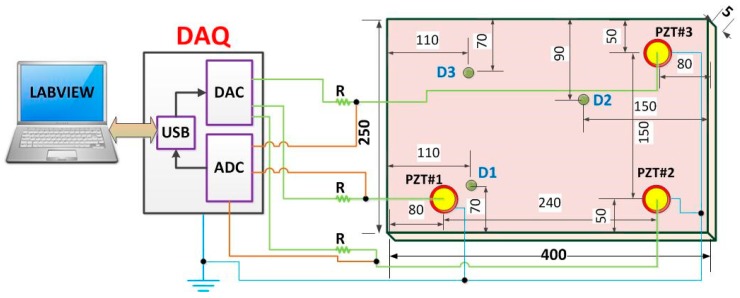
Acquisition system along with the plate, PZT and damage positions (dimensions in mm).

The experiments were carried out on an aluminum plate with dimensions of 400 mm × 250 mm × 5 mm. The plate was suspended using fishing lines in both tips to simulate free-free boundary conditions. Posteriorly, three piezoelectric diaphragms (called PZT#1, PZT#2, and PZT#3) with diameters of 12 mm were used, which had active elements of type P-7 PZT ceramics (Murata Electronics). These diaphragms were bonded (using 3M Scotch-Weld Epoxy Adhesives DP460 Off-White) to the plate at three different positions (Figure 5). According to a previous study [46], piezoelectric diaphragms (also known as buzzers) have a simple circular construction consisting of a brass plate onto which a piezoelectric ceramic disc is fixed. These acoustic components are readily obtained and inexpensive, which make them attractive for other applications apart from producing sound. Following this, a chirp signal sweeping from 20 kHz to 110 kHz with an amplitude of 3 V was used to excite the set PZT/structure. Although many authors have mentioned that the real part of EMI in a frequency ranges from 20 kHz up to 40 kHz [6,7], the best frequency band of the EMI signature for higher sensitivity and repeatability in detecting structural damage depends on several issues, such as geometry, mass, boundary conditions, and other structure characteristics [47]. Furthermore, the frequency range was chosen in this present study because in high frequencies, the structure suffers less interference of global conditions when compared to low vibration modes [3]. Likewise, studies have shown that the variation in terms of voltage amplitude used for exciting PZTs is insignificant when applied to the EMI-based methods [48]. In order to limit the electric current through the PZT patch, the resistor R was set to 1 kΩ.

**Figure 6:**
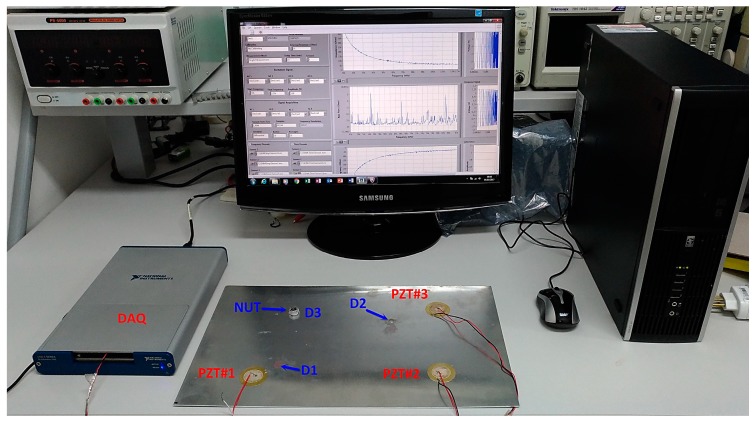
Experimental apparatus including: the aluminum plate, containing three PZT patches, DAQ, and computer running the acquisition software.

The data were recorded using the acquisition system proposed in a previous study [49]. This system manages a multifunctional Data Acquisition (National Instruments DAQ) device model USB-6259 (Figure 6). All of the configuration parameters, such as sample rate, frequency range, and voltage amplitude, are easily set at the acquisition software, which was developed in LabVIEW [49]. The acquisition software provides the real, imaginary and module of the EMI. Furthermore, the software provides the time for the response of the set PZT/structure. PZT response signals were sampled at a rate of 1 MS/s. Further details about the sampling method and how both the FRF and EMI are computed can be obtained from a previous study [49]. It is important to mention that PZT patches were connected to the acquisition system via copper wires. The wires were soldered with the PZTs using a tin–silver–copper solder. It is important to mention that throughout all of the experiments, the environmental temperature was maintained at a constant 22 °C. All of the procedures were conducted based on the EMI technique [7,16,20]. It is important to highlight that each PZT patch acts as a sensor and actuator simultaneously (EMI-based technique).

The responses of the pristine structure at each sensor were first acquired in order to form the baseline (BL). Posteriorly, a new cycle considering the pristine structural condition was carried out to form the healthy structural condition (H). Finally, a small nut with diameter of 12 mm and height of 7 mm (about 30 g) was separately bonded (using 3M Scotch-Weld Epoxy Adhesives DP460 Off-White) to the structure at three different positions in order to form features of the damaged structure (Figure 6). Damage positions were called as D1, D2, and D3. The total impedance signals for each PZT sensor was considered, with each structural condition being 60. The interval of the time between two consecutives samples was 30 s. These signals are smoothed by SG and derivatives before computing Euclidean Distances (ED) for the formation of datasets.

### 3.2. Forming Datasets

This paper proposes a new way of forming datasets in the SHM field. The acquired data set was divided into six sub-data sets. The first was formed after all impedance signals in the time-domain, having been smoothed by the Savitzky–Golay (SG) filter. From all smoothed signals, Euclidean Distances (EDs) were computed as follows (Figure 4):

(12) $$\mathrm{ED}\left(\mathrm{BL},U\right)=\sqrt{\sum_{j=1}^{n}{\left({\mathrm{BL}}_{j}-U_{j}\right)}^{2}}.$$

where BL is the SG-smoothed signal for the baseline, U is the SG-smoothed signal for unknown structural condition, and n is the total of samples. The structural response signals were smoothed by a 10th polynomial order with frame length of 501 (SG setup). The second dataset was formed after applying the first derivative over the SG-Smoothed signals (Equation (11)). From these smoothed signals, EDs were also computed by using Equation (12). We named this dataset SGFD. The third one is performed similarly. However, we computed the second derivative (Equation (11)) over the SG-Smoothed signals before computing EDs (named as SGSD).

The last three datasets were instead formed using the real part of the EMI in the frequency domain. Impedance signals (frequency domain) were smoothed by SG, followed by computation of the first and second derivatives. EDs were computed from the obtained signals after applying the derivatives. Similar ones were obtained for the real part of the EMI (frequency domain) for each PZT sensor. We split each dataset, with 60% being used for training and 40% for testing. Each dataset has 60 samples for each structural condition (H, D1, D2, and D3), with a total of 240 samples. These datasets were used as inputs for the SFAN/PNN classifiers. Within the dataset, we used 144 and 96 samples for training and testing, respectively.

### 3.3. Damage Detection Phase

In order to guarantee the accuracy and repeatability for the proposed method, the damage detection phase uses two different neural networks: the Probabilistic Neural network (PNN) and the Simplified Fuzzy ARTMAP Network (SFAN). One PNN or SFAN network is created for each PZT sensor, thus resulting in a total of three networks. Each neural network is separately fed with one of the datasets described above. For both training and testing procedures, each structural condition (H, D1, D2, and D3) was labelled as 1, 2, 3, and 4, respectively (Figure 4). Hence, the method is responsible for classifying four different categories. The FAN parameters are set as follows: the choice parameter (α = 0.25), the training rate (β = 1), the vigilance parameter (ρ = 0.78), and the match tracking (ε = 0. 85). The spread constant (σ) for PNN was set to 0.1. The choice of those parameters was made based on reference [36].

## 4. Experimental Results

**Figure 7:**
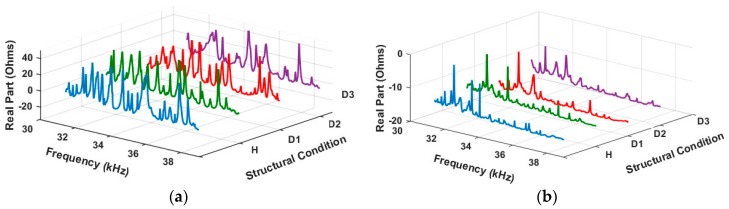
Electromechanical Impedance (EMI) signatures for various structural conditions (H, D1, D2, and D3): (**a**) PZT#1; and (**b**) PZT#2.

In order to evaluate the proposed methodology, this section presents the experimental results. Firstly, Figure 7a depicts the impedance signatures for PZT#1, while Figure 7b depicts the related signals for PZT#2: both signals for the healthy condition (H) and for three different damages (D1, D2, and D3). For the purpose of brevity, only the signals for the real part of the EMI (frequency domain) are shown. It is important to note that the differences between the signals representing different structural conditions are barely perceptible. As presented in this approach, signal processing techniques are an excellent way to improve such differences. This results in clear and reliable damage identification.

**Figure 8:**
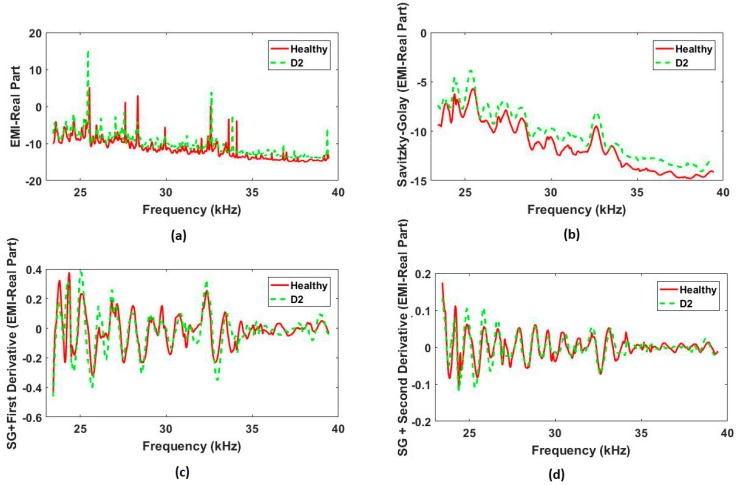
Real part of the EMI signatures considering two structural conditions (H and D2) for PZT#2: (**a**) raw EMI; (**b**) EMI signals smoothed by Savitzky-Golay (SG); (**c**) EMI signals smoothed by SG, followed by application of the first derivative; and, (**d**) EMI signals smoothed by SG, followed by application of the second derivative.

Another important issue to be analyzed relates to the signal variation after applying SG and its respective first and second derivatives. Hence, Figure 8a shows the raw EMI (real part) signature for PZT#2 when considering two different structural conditions: H and D2. For the purpose of brevity, only the signals for the real part of the EMI (frequency domain) are shown. As observed, the difference between the signals is clear. Posteriorly, both of the signals were smoothed by SG and the obtained results are presented in Figure 8b. As seen, the difference between the curves seems to be much bigger than for EMI, which may result in an improvement in terms of success rates when the damage detection is being performed by the SFAN/PNN algorithms.

Figure 8c,d shows the real part of the EMI for PZT#2 after application of SG, followed by the application of the first and second derivatives, respectively. For both of the cases, the difference between the signatures for H and D2 seems to be smaller than for only signals that are smoothed by SG (Figure 8b). However, it is difficult to visually judge these differences. Hence, this approach proposes the use of PNN/SFAN-based method in order to enhance the damage detection. The PNN/SFAN results are presented next.

### 4.1. Study Case 1: Real Part of the EMI

To identify structural damage, datasets were used as inputs for the neural networks. Three PNN and three SFAN (one for each PZT) were implemented for the analysis of structural conditions. All these networks were built with the same architecture, since they were all intended to the same purpose (monitoring the structural damage in an aluminum plate). Hence, the first analysis is depicted in Table 1. The results are for both PNN- and SFAN-based methods with consideration of datasets formed from the smoothed signals by SG, first (SGFD) and second (SGSD) derivatives. It is important to mention that SG and derivatives were applied to the real part of the EMI (frequency domain). The results are compared with PNN- and SFAN-based methods, which were performed by computing the Euclidean Distances (ED) directly from EMI signals instead (methods were proposed in reference [36]). All of the methods are implemented to the same datasets and same conditions in order to obtain a fair comparison.

As shown in Table 1, the results have indicated improved success rates for two cases (PZT#2 and PZT#3) when using PNN along with SG. Similar results were obtained for the SGFD. When considering the SGSD method, only one case (PZT#2) demonstrated any improvement, while others presented very poor results. As shown in Table 1, SFAN has shown better performances (100%) for all four cases. Analyzing only the improved cases, it is clear that the increases in the hit rates were quite significant for the enhanced methods. Overall, the SGFD method seems to be more precise in identifying structural damage in the frequency domain. This result is perceived through the analysis of the differences between the EMI signatures for the healthy and damaged conditions (Figure 8c). Unfortunately, none of the investigated methods have shown overall improvement of the accuracy of prediction.

### 4.2. Study Case 2: Time-Response Signals

The second analysis considers the same aforementioned conditions. However, the SG and derivatives were applied to the impedance signals in the time domain. It is important to clarify that the damage detection only considers the time domain response (voltage signals) without considering FRF, electromechanical impedance, or Laplace/Fourier Transform. For that, the PZT transducer is excited, and only its time response signal (voltage) is directly used to compute Euclidean Distances. It is important to remark that even analysis of the excitation signal has not shown that the time response signals are correlated with the electromechanical impedance (since it is guaranteed that the excitation input is kept constant) [37,50]. Hence, working on the time domain simplifies and speeds up the damage identification because the EMI is not computed, which by itself, is a great advantage in terms of processing time [37,50]. In this sense, Table 2 depicts the results for both PNN and SFAN. The methods used are outlined in reference [36]. All of the methods are also implemented with consideration of the same datasets and same conditions in order to obtain a fair comparison.

As observed from Table 2, the methods based on SG and SGSD had significant performance enhancement for both PNN and SFAN when compared with the ED-based method [36]. Unlike the method based on the frequency domain, the SGFD method showed poorer performance when considering time domain analysis. It is important to note that only the PZT#2 did not have better performance for the SFAN-based method, while only PZT#3 had improved performance for the PNN-based method (SGFD). It is remarkable that PNN obtained the same hit rates for the SG and SCSD methods. Similar results are also shown for SFAN. As expected, SFAN performed better than PNN, as shown in reference [36]. Clearly, it is an undisputed fact that the methods presented in this approach were effectively able to successfully identify various structural conditions with much higher hit rates for both PNN and SFAN.

## 5. Discussion

**Figure 9:**
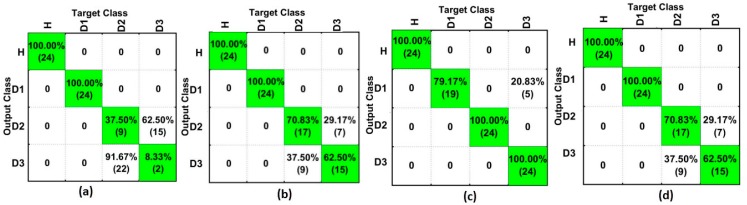
Results for PZT#1 and SFAN (time domain) applied to: (**a**) Euclidean Distance (ED) [35]; (**b**) Savitzky-Golay (SG); (**c**) SG + First Derivative (SGFD); and, (**d**) SG + Second Derivative (SGSD).

In order to estimate the accuracy of analysis, the confusion matrices are presented. Firstly, Figure 9 depicts the comparison between ED (Figure 9a), SG (Figure 9b), SGFD (Figure 9c), and SGSD (Figure 9d) for PZT#1 when considering the SFAN classifier. Henceforth, all of the presented results relate to the SFAN-based method. As observed from Figure 9, the structure with no damage was correctly classified in all experiments. Likewise, the structure with damage was never confused with the structure with no damage. This means that the errors that appear in the classification are merely due to a mistake in the quantification of the damage. Furthermore, D1 structural condition was detected 100% correctly for three methods, including the reference (Figure 9a). This result was anticipated because D1 was placed near to PZT#1. It is clearly evident that the results for D2 and D3 (located further away from PZT#1) were significantly improved when compared with the ED [35]. The method based on SGFD detected all of the structural conditions with a hit rate of over 79%. Overall, the proposed methods (SG, SGFD, and SGSD) performed better when compared with the ED-based method [36].

**Figure 10:**
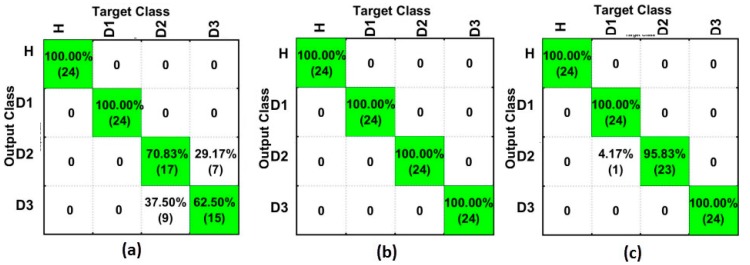
Results for the SFAN-based method (time domain) considering only Savitzky–Golay (SG) for: (**a**) PZT#1; (**b**) PZT#2; and, (**c**) PZT#3.

Figure 10 shows the results for the SG-based method for PZT#1 (Figure 10a), PZT#2 (Figure 10b) and PZT#3 (Figure 10c). As observed, PZT#2 detected all the structural conditions with success rates of 100%. Likewise, PZT#3 obtained very precise results. PZT#1 obtained a hit rate of 100% for D1, although it had poorer results for D2 and D3. Regardless, this highlights that the methods all had excellent performance in identifying all the different structural conditions. These poor results are justified since D2 and D3 are placed further away from PZT#1 (see Figure 6).

**Figure 11:**
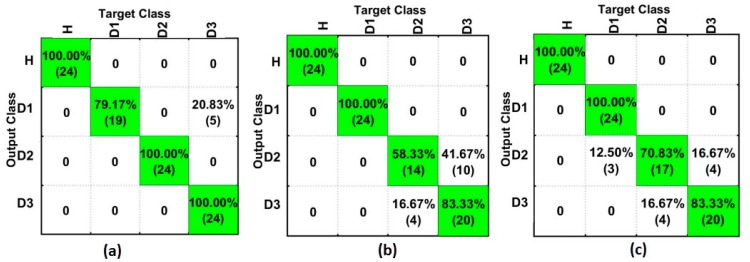
Results for the SFAN-based method (time domain) considering only Savitzky–Golay + First Derivative (SGFD) for: (**a**) PZT#1; (**b**) PZT#2; and, (**c**) PZT#3.

Figure 11 depicts the results for the SGFD-based method for PZT#1 (Figure 11a), PZT#2 (Figure 11b), and PZT#3 (Figure 11c). As seen, PZT#1 had significant improvement if compared to the results presented in Figure 10a. All of the structural conditions were detected with a hit rate of over 79%, which is considered to indicate great performance. On the other hand, PZT#2 and PZT#3 had slightly worsened performance. However, their results are still over 70%, excepting for PZT#2 and D2, which is considered good performance in the field of neural networks. Once again, it can be considered that the results obtained all showed satisfactory performance in identifying all the different structural conditions.

**Figure 12:**
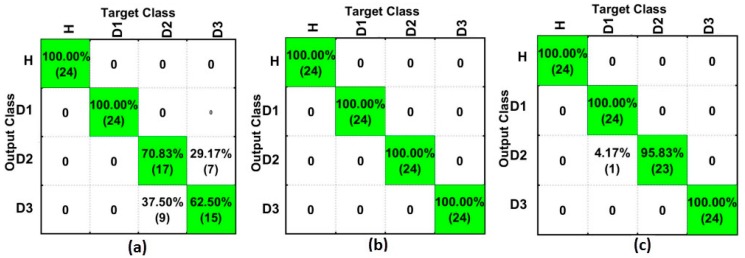
Results for the SFAN-based method (time domain) considering only Savitzky-Golay + Second Derivative (SGSD) for: (**a**) PZT#1; (**b**) PZT#2; and, (**c**) PZT#3.

Figure 12 shows the results for the SGSD-based method for PZT#1 (Figure 12a), PZT#2 (Figure 12b), and PZT#3 (Figure 12c). As observed, PZT#2 detected all structural conditions with success rates of 100%. Likewise, PZT#3 obtained very precise results. PZT#1 obtained a hit rate of 100% for D1, although it provided poorer results for D2 and D3. Regardless, we can consider that the obtained results indicate excellent performance in identifying all of the different structural conditions. In short, the presented results for SGSD are quite similar to those presented for SG (Figure 10).

**Figure 13:**
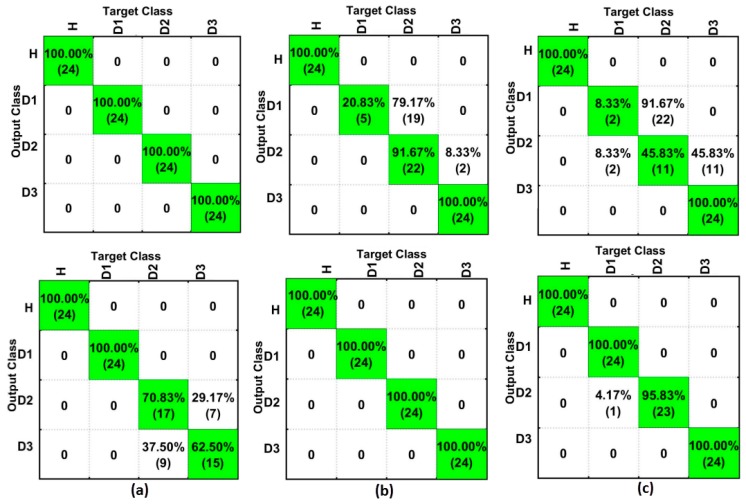
Comparison among the best obtained results for SFAN in the frequency domain (top), and SFAN in the time domain (bottom), for: (**a**) PZT#1; (**b**) PZT#2; and, (**c**) PZT#3.

Finally, Figure 13 shows a comparison between the best results for the analysis in the frequency domain (top) and time domain (bottom), considering only the SFAN-based method for PZT#1 (Figure 13a), PZT#2 (Figure 13b) and PZT#3 (Figure 13c). As observed, PZT#2 and PZT#3 had substantial improvement in the time domain analysis, with a hit rate of over 90% for the best analyzed case (D1). Furthermore, PZT#1 detected H and D1 structural conditions with success rates of 100% when considering the time domain analysis. On the other hand, PZT#1 had better performance for the frequency domain-based method when considering the damage in D2 and D3. Interestingly, the analysis conducted in the time domain has shown an improved accuracy of SFAN-based methods (Figure 13). This result is consistent with those in reference [35]. It is important to highlight that the damage detection in the time domain was carried out without considering the Frequency Response Function (FRF) or inverse Laplace/Fourier Transform, which by itself is a great advantage in terms of computational efforts. It simplifies and speeds up the damage detection in real applications.

In particular, the data processing time and the accuracy of prediction are the major indicators of a SHM system. The cost of a SHM system will increase with an increasing number of sensors in the SHM system. As a consequence, the number of data points will be increased by thousands, and the processing time will be substantially increased. As such, an optimized “low cost” and “efficient” SHM system is urgently required in every engineering field. Beyond accuracy rates, the time consumptions for both testing and training phases were also investigated for PZT#2, when only considering the SGFD method. Training and testing times for SFAN were 0.126 s and 0.0079 s, while these were 1.672 s and 0.6742 s for the PNN, respectively. The results showed that the SFAN-based method consumed less time for both training and testing phases when compared with PNN. This result was expected, as it is consistent with those from previous studies [36,37]. As a consequence, the SFAN-based method seems to be very reliable for implementing real-time SHM systems, especially for the testing phase by using an ordinary laptop. All of the time measurements were carried in a Laptop running Windows 7 Ultimate. The laptop configuration consists of an Intel Core i3-2.2 GHz with 4 GB of RAM. Detailed analysis of the time consumption for PNN- and SFAN-based methods can be read in references [36,37].

It is important to note that the use of SG and its derivatives for the detection of the changes within EMI signatures of a structure can be affected by low noise, environmental interferences, and low vibration modes. Those problems were the subject of a previous study [51]. Future research will be undertaken in including the SFAN method in a microcontroller or in another low-cost hardware, as suggested as a previous study [52]. Furthermore, future work should focus on using the optimization algorithm, such as Particle Swarm Optimization (PSO), to enhance the accuracy rate. The idea is to use PSO for selecting the optimal setup parameters for SFAN (choice parameter (α), training rate (β), vigilance parameter (ρ), and match tracking (ε)) to improve the classifier performance, thereby maximizing the success rate of the classified results [37]. Furthermore, future investigations should use structures made up of composite materials order to investigate incipient damage and its progression. Furthermore, future research will be undertaken to evaluate the accuracy of the proposed method for randomly initiated defects and to establish the outcomes from having two or more areas of damage at the same time. Another interesting point to be addressed is the evaluation of different types and sizes of damage [10,12,15].

Comparing the results obtained here with that of previous research in literature is quite difficult due to the lack of many available approaches focusing on a similar scenario, which can lead to unfair comparison. There are only three approaches that focus on SFAN and EMI available in published research work [35,36,37]. They have investigated the progression of structural damage on composite materials.

## 6. Final Remarks

This work introduced an improved method to detect damage in structures by exploiting the EMI technique along with two different neural networks: SFAN and PNN. Three different methods were used to form datasets, which were used as inputs to the neural networks: Savitzky–Golay (SG), Savitzky–Golay with the first derivative (SGFD), and Savitzky–Golay with the second derivative (SGSD). Furthermore, the analyses were carried in both time and frequency domains. As an example, the method was applied to an aluminum plate. Three PZT sensors were bonded to the plate in order to excite the plate and obtain structural responses. The results were presented in terms of success rates from the confusion matrices analysis.

In summary, the comparative analysis has shown that the SFAN method has shown relatively enhanced performances in the detection of damage in a structure when compared to PNN-based methods. Furthermore, the methods based on SFAN incorporated with time domain analysis have significantly enhanced the damage detection ability. As observed from these results, the proposed method was also successfully able to identify various structural conditions with higher rates of accuracy when using the SG, SGFD, and SGSD. After further investigating the results, the structure with no damage was correctly classified in all of the experiments. Likewise, the structure with damage was never confused with the structure with no damage. Furthermore, each structural condition was mostly detected with an overall success rate of over 70%, which is significantly higher than state-of-the-art approaches. Moreover, this approach yields in an improvement of 90% when considering the best case scenario. The results show excellent performance within the field of neural networks.

The outcomes of this study have shown that the efficiency, accuracy, and the robustness of the proposed method in detecting damages in structures. The results conclusively confirmed that the proposed approach can effectively lead to an increase in safety and reduce maintenance costs in SHM systems. It is important to note that temperature variation is a classical issue in SHM systems when using PZT sensors, which was negligible in this present analysis.

## Figures and Tables

**Table 1:** Success rates for Probabilistic Neural Network (PNN) and Simplified Fuzzy ARTMAP (SFAN) methods for the real part of the EMI (frequency domain).

	PNN [36]	PNN			SFAN [36]	SFAN		
Sensor	ED	SG	SGFD	SGSD	ED	SG	SGFD	SGSD
PZT#1	75%	67.71%	50%	48.95%	71.87%	100%	46.87%	50%
PZT#2	70.83%	75%	100%	100%	84.37%	78.12%	100%	100%
PZT#3	77.08%	78.12%	78.12%	59.37%	75%	63.54%	83.33%	47.91%

**Table 2:** Success rates for PNN and SFAN methods for impedance signals in the time domain.

	PNN [36]	PNN			SFAN [36]	SFAN		
Sensor	ED	SG	SGFD	SGSD	ED	SG	SGFD	SGSD
PZT#1	75%	75%	50%	75%	61.45%	83.33%	94.79%	83.33%
PZT#2	96.87%	100%	75%	100%	98.95%	100%	85.41%	100%
PZT#3	70.83%	98.95%	85.41%	98.95%	77.08%	98.95%	88.54%	98.95%
